# Editorial: COVID-19 Related Kidney Disease: From Epidemiology to Clinical Management

**DOI:** 10.3389/fmed.2022.956409

**Published:** 2022-06-28

**Authors:** Marco Fiorentino, Vincenzo Cantaluppi, Ravindra L. Mehta

**Affiliations:** ^1^Department of Emergency and Organ Transplantation, Nephrology Dialysis and Transplantation Unit, University of Bari, Bari, Italy; ^2^Nephrology and Kidney Transplantation Unit, Department of Translational Medicine, University of Piemonte Orientale, Novara, Italy; ^3^Division of Nephrology, Department of Medicine, University of California, San Diego, San Diego, CA, United States

**Keywords:** COVID-19, kidney disease, dialysis, acute kidney injury, vaccination, kidney transplant

Severe acute respiratory syndrome coronavirus 2 (SARS-CoV-2) was first described in China in December 2019, but rapidly spread around the world causing the coronavirus disease 2019 (COVID-19) and the current global pandemic. The clinical spectrum is broad, ranging from asymptomatic patients or with mild upper respiratory tract infection to patients requiring admission to intensive care unit (ICUs). Although, the pulmonary manifestations of COVID-19 are most prominent, the development of acute kidney injury (AKI) is now recognized as a common complication of the disease and is often evident since hospital admission (1, 2). This Research Topic aims to highlight the epidemiological aspects of renal dysfunction, including the incidence and risk factors of COVID-19 associated AKI in critically ill patients, as well as novel therapeutic options and approaches for patients with SARS-CoV-2 infection.

Several reports indicate significant rates of AKI, particularly in the intensive care setting, with the need of kidney replacement therapy (KRT) in up to 45% of ICU patients (3). As recently reported by a joint survey of SIN/SIAARTI societies that recorded about 19,000 cases of COVID-19 in Italy, only 43% of clinicians routinely used KDIGO criteria with a possible underestimation of AKI incidence (4). Cai et al. herein performed a systematic review and meta-analysis to explore and summarize the several risk factors for AKI reported in the literature. The meta-analysis included 38 studies with 42,779 patients and showed that male gender, older age, the presence of specific comorbidities such as smoking, obesity, hypertension, diabetes, pneumopathy, cardiovascular disease, cancer, chronic kidney disease (CKD) were significant risk factors for AKI; moreover, the need of mechanical ventilation and the use of vasopressors were strongly associated with AKI.

Mortality among hospitalized patients with COVID-19-associated AKI is higher than for those without AKI (5, 6). Moreover, low rates of renal recovery and the progression to chronic kidney disease (CKD) have been described in the post-acute COVID-19 disease. Regolisti et al. conducted a retrospective study in 115 critically ill patients with COVID-19 to explore whether the severity of clinical conditions at ICU admission affects the impact of AKI on 28-day mortality. Interestingly, when stratifying the study cohort by SOFA score (lowest, middle, and highest tertiles), the onset of AKI was strongly associated with mortality particularly in patients with less severe conditions at admission (lowest and middle SOFA tertiles). Thus, AKI development is associated with mortality not only in the context of severe disease and multiorgan failure, but it may serve as a disease modifier in less critical patients contributing to adverse clinical outcomes.

Several potential mechanisms have been described through which SARS-CoV-2 infection might determine direct and indirect effects on the kidney, inducing renal dysfunction and/or failure (7). Henry et al. performed a prospective multicenter study investigating the role of complement as well as inflammatory and thrombotic parameters in 131 COVID-19 patients requiring hospitalization at one US and two Hungarian centers. The authors showed a significant complement over-activation and consumption in patients who developed severe AKI and required RRT during hospitalization. C3a/C3 ratio was increased in groups developing severe AKI (3.29 vs. 1.71; *p* < 0.001) and requiring RRT (3.42 vs. 1.79; *p* < 0.001) in each cohort. In addition, the decrease in alternative and classical pathway activity, C4 consumption and C3a elevation were also reported in patients with severe AKI, suggesting this pathway as a promising therapeutic target for thrombotic microangiopathy in COVID-19 patients.

As previously shown, CKD and other comorbidities such as diabetes mellitus, hypertension, and obesity have been linked to mortality in patients with COVID-19 (8). Similarly, patients with end stage kidney disease (ESKD) on chronic dialysis or with kidney transplant recipients (KTR) are typically identified as categories at high risk for severe course of the COVID-19 disease (9, 10); an optimal clinical and therapeutic management of these patients is critical in improving clinical outcomes. Despite a great debate concerning the presence of the so-called “cytokine storm” in COVID-19 patients, in this Research Topic, Infante et al. reported the positive effect of tocilizumab (a humanized monoclonal antibody against IL-6 receptor) administration in a kidney transplant recipient with proven SARS-CoV-2 pneumonia and ARDS who developed acute graft dysfunction. The rationale for the use in this setting was to control the activation of the immune system with a consequent cytokine storm. The days after a single administration of tocilizumab, the patient presented a significant clinical (increased diuresis and oxygen saturation), laboratory (reduced white blood cells count, C-reactive protein, IL-6) and radiological (reduction of pulmonary opacities) improvements, supporting the importance of immunomodulation in COVID-19 disease, particularly in this specific subgroup of patients. Furthermore, AKI may worsen the inflammatory state of these critically ill patients, since several inflammatory mediators are both eliminated and produced by kidney resident cells (e.g., tubular epithelial cells) that are immunologically active.

Finally, the introduction of vaccination program had completely changed the overall course of COVID-19 disease, with a prevalence of low severity cases and, consequently, the reduction of ICU and hospital pressure in the third and fourth waves of pandemic. However, descriptive studies have assessed the serological response of specific patient groups to vaccination, and they have revealed impaired seroconversion rates in patients living with ESKD, solid-organ transplants and in patients receiving immunosuppression, compared to the general population (11). In this Research Topic, we report the experience of Carruthers et al., who collected data on seroconversion response rates to COVID-19 vaccination in a cohort of 81 patients with ANCA-associated vasculitis and renal involvement. In this study cohort, seroconversion after the first dose was achieved in only 63.6% of patients, while it reached 82.6% after the second dose. Similarly, Agur et al. conducted a prospective cohort study to analyze the seroconversion rate and its longevity at 6 months following the second dose of the BNT162b2 vaccine in 104 patients on chronic hemodialysis, compared with a control group of 84 participants. The authors reported that, despite the early humoral response, the 6-month seropositivity rate in patients on hemodialysis was significantly lower than in the control group (79.8 vs. 98.8%, *p* < 0.001). Both these reports add to the growing evidence of reduced seroconversion in response to vaccination in patients with renal disease of any cause and strongly support the need for identifying and targeting booster vaccination programs to vulnerable patient cohorts. In addition, AKI may lead to the development of several alterations of adaptive immunity, in particular a defective T and B cell response directed to SARS-CoV-2.

In summary, the manuscripts in this Research Topic Series provides a contemporary view of the emerging knowledge of kidney dysfunction in the setting of SARS-CoV-2 infection. We recognize the continuing need to learn from the worldwide experience, given the unabated waves of the pandemic across the world. We hope readers will find these articles helpful as they manage their patients with this insidious disease complicated by AKI development.

## Author Contributions

MF, VC, and RM contributed to the study conception and design of the Research Topic. All authors contributed to the article and approved the submitted version.

## Conflict of Interest

The authors declare that the research was conducted in the absence of any commercial or financial relationships that could be construed as a potential conflict of interest.

## Publisher's Note

All claims expressed in this article are solely those of the authors and do not necessarily represent those of their affiliated organizations, or those of the publisher, the editors and the reviewers. Any product that may be evaluated in this article, or claim that may be made by its manufacturer, is not guaranteed or endorsed by the publisher.
